# Transperineal Free-Hand Prostate Fusion Biopsy with AI-Driven Auto-Contouring: First Results of a Prospective Study

**DOI:** 10.3390/cancers17142381

**Published:** 2025-07-18

**Authors:** Marco Oderda, Giorgio Calleris, Alessandro Dematteis, Alessandro Greco, Alessandro Marquis, Giancarlo Marra, Umberto Merani, Alberto Sasia, Alessio Venturi, Andrea Zitella, Paolo Gontero

**Affiliations:** Division of Urology, Department of Surgical Sciences, Molinette Hospital, University of Turin, 10126 Torino, Italy; giorgio.calleris@unito.it (G.C.); alessandro.dematteis@unito.it (A.D.); alegreco72@yahoo.com (A.G.); alessandro.marquis@gmail.com (A.M.); giancarlo.marra@unito.it (G.M.); umberto.merani@unito.it (U.M.); alberto.sasia@unito.it (A.S.); alessio.venturi@unito.it (A.V.); andrea.zitella@unito.it (A.Z.); paolo.gontero@unito.it (P.G.)

**Keywords:** prostate biopsy, fusion, transperineal, free-hand, esaote, auto-contouring, AI

## Abstract

**Background**: prostate fusion biopsies are key in the diagnosis of prostate cancer (PCa); however, the fusion imaging system is not always user-friendly or reliable. The aim of this study was to assess the feasibility, accuracy, and effectiveness of transperineal fusion biopsies performed with a novel fusion imaging device equipped with AI-driven auto-contouring. **Methods**: data from 148 patients who underwent MRI-targeted and systematic prostate fusion biopsy with UroFusion (Esaote) were prospectively collected. All biopsies were performed in-office, under local anaesthesia. **Results**: cancer detection rate was 64% overall and 56% for clinically significant PCa (csPCa, ISUP ≥ 2). PCa was detected in 35%, 65% and 84% of lesions scored as PI-RADS 3, 4 and 5, respectively. Outfield positive systematic cores were found in the contralateral lobe in one third of cases. Median device-time to obtain fusion imaging was 5 min and median biopsy duration was 15 min. Median difference in volume estimation between ultrasound and MRI auto-contouring was only 1 cc. Detection rate did not differ between experienced and novice, supervised users. **Conclusions**: in this initial prospective experience, fusion biopsies performed with UroFusion AI-driven auto-contouring system appeared time-efficient, accurate, well tolerated, and user-friendly, with comparable outcomes between experienced and novice users. Systematic biopsies remain highly recommended given the non-negligible rates of positive outfield cores.

## Simple Summary

The present study explored the role of a novel device for fusion imaging, equipped with AI-driven auto-contouring, to perform prostate fusion biopsies. We found that the device was accurate for targeting MRI lesions, with good detection rates in line with previous findings in the literature. The procedure with UroFusion appeared time-efficient, user-friendly, and well-tolerated, allowing good outcomes with novice users. Fusion imaging was accurate, with a median difference of 1 cc between the volumes estimated at ultrasound and MRI.

## 1. Introduction

Transperineal biopsy with MRI-targeted and systematic sampling has become the gold standard for the diagnosis of prostate cancer (PCa), recommended by the current guidelines of the European Association of Urology [1]. Fusion imaging guided by software seems to achieve better outcomes as compared to a cognitive approach [2,3], despite the fact that no significant differences in clinically significant PCa (csPCa) detection rates have been detected in a recent meta-analysis focusing on comparative research [4]. To be effective, fusion imaging systems should be accurate in the alignment of MRI and ultrasound sequences but also user-friendly and not excessively time-consuming. Furthermore, a certain degree of experience in MRI reading is required for the operator.

In recent years, advancements in the field of artificial intelligence (AI) have led to improvements in various tasks during prostate biopsy, such as prostate contouring, segmentation, and tumour detection [5]. AI has the potential to speed up the workflow of fusion biopsy while reducing inconsistencies between observers, and finally, improving diagnostic accuracy [6]. To date, the incorporation of AI algorithms in fusion biopsy systems is still limited. The aim of the present study was to assess the accuracy and effectiveness of transperineal fusion biopsies performed under local anaesthesia with a novel fusion imaging device equipped with AI-driven auto-contouring.

## 2. Materials and Methods

### 2.1. Study Population

Data from 148 patients who underwent MRI-targeted and systematic prostate biopsy with Esaote MyLab 9 (Esaote, Genova, Italy) between January 2024 and March 2025 were prospectively collected at our tertiary referral centre. All patients had at least one MRI lesion scored as PI-RADS score ≥ 3. The study was conducted according to the Helsinki declaration and all patients signed an informed consent for data collection. The study was approved by the “A.O.U. Città della Salute e della Scienza” ethics committee (N. 0040478/2020).

### 2.2. MRI and Biopsy Technique

**Figure 1:**
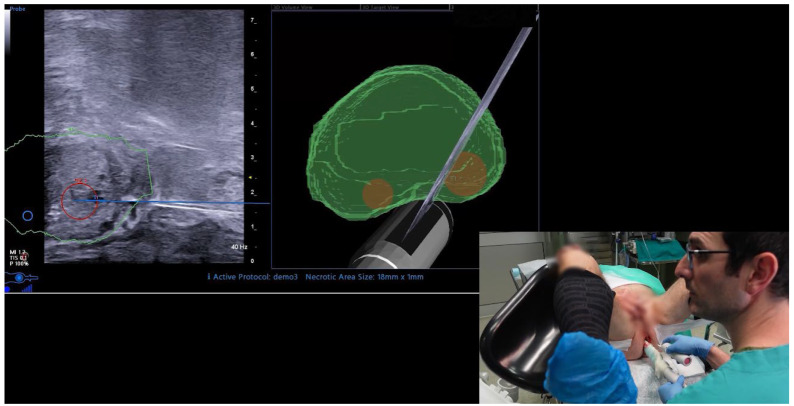
Fusion biopsy performed with AI-driven UroFusion auto-contouring software.

Prebiopsy MRIs were performed using a 1.5 T or 3 T scanner and consisted of multiplane T1- and T2-weighted imaging, diffusion-weighted imaging (DWI), and dynamic contrast enhancement according to the European Society of Urogenital Radiology guidelines. Scans were reviewed and scored using the PI-RADS v.2.1 protocol. MRI-targeted fusion biopsies were performed in-office under local anaesthesia with an Esaote MyLab X9 (Esaote, Genova, Italy) device equipped with AI-driven UroFusion auto-contouring software (Figure 1). Local anaesthesia consisted of peri-prostatic injections of lidocaine 2% 1 fl with 20 cc saline solution, or other types of local anaesthetics at physician’s preference. Biopsies were performed with a free-hand technique (previously published by our group [7]). A minimum of 3 targeted cores were taken per target, followed by the systematic mapping of the peripheral zone, with different sampling of the apex, mid-gland, and base of the prostate, both in the lateral and paramedian zones. Typically, 12 systematic cores were taken, with variations according to the clinical case. Each biopsy core was separately sent for pathologic examination, specifying the location of the sampling.

Visual Analog Scale (VAS) score was collected at the end of the procedure, taking care to explain to the patient how the score was set depending on the severity of the pain.

### 2.3. UroFusion Software

**Figure 2:**
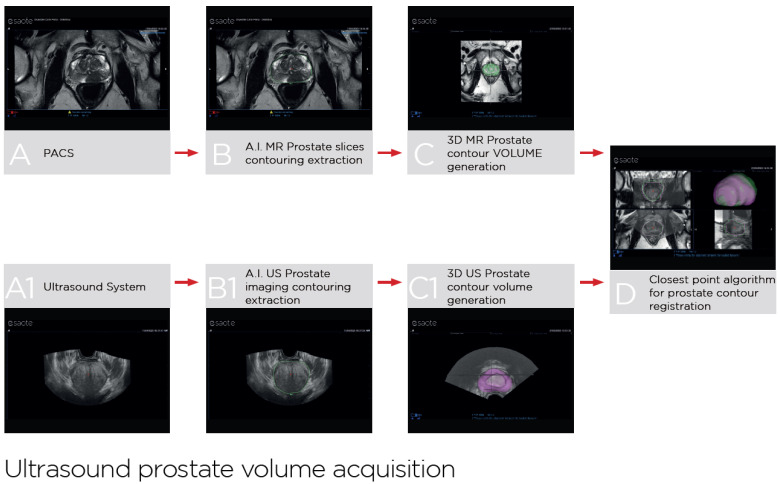
UroFusion automated prostate segmentation and fusion workflow: AI-driven automatic prostate contouring on MRI T2-weighted DICOM sequences and ultrasound volume acquired with a panoramic sweep of the gland by the probe using a specific movement transducer, leading to fused image registration. (**A**): T2 MRI axial view; (**A1**): US axial acquisition; (**B**): AI-driven prostate contouring on MRI; (**B1**): AI-driven prostate contouring on US; (**C**): MRI prostate contour volume generation; (**C1**): US prostate contour volume generation; (**D**): fused registration of MRI and US prostate volumes.

UroFusion (https://www2.esaote.com/ultrasound/clinical-solutions/urology-ultrasound/, accessed on 15 July 2025) is a dedicated software integrated into the latest generation of Esaote ultrasound systems (Esaote, Genova, Italy), which automatize prostate segmentation on MRI T2-weighted DICOM sequences and ultrasound volume acquired with a panoramic sweep of the gland by the probe using a specific movement transducer. AI-powered algorithms align the MRI and ultrasound datasets by matching the prostate contours, minimizing the distance between corresponding points and compensating for deformations and rotational discrepancies. This results in real-time and operator-independent fusion imaging (Figure 2).

### 2.4. Endpoints and Statistical Analyses

The endpoints of study were to evaluate (1) the detection of any PCa and csPCa, defined as ISUP grade group 2 or greater; (2) the duration of the fusion imaging, intended from the import of DICOM images to the auto-contouring of the prostate, and the duration of the biopsy itself, till the exit of the patient; (3) the correlation between ultrasound and MRI volumes generated by automatic segmentation; (4) the pain VAS score at the end of the procedure.

Continuous data were reported as means and standard deviation and categorical parameters were shown as counts/percentages. Pearson chi-square test was used to compare categorical variables. Statistical significance was set at two-sided *p* < 0.05. Statistical analyses were performed with SPSS version 29.0 (IBM Corp., Armonk, NY, USA).

## 3. Results

Baseline patient characteristics are shown in Table 1. Median age, PSA, and PSA-density were 69 years (IQR 64–76), 6.5 ng/mL (IQR 3.9–9.1), and 0.13 (IQR 0.06–0.18), respectively. Biopsy-naïve patients represented the 84% of our cohort. A suspicion in digital rectal examination was found in 34% of cases, while only 11% had a suspect extracapsular extension at MRI. The median prostate volumes estimated at preoperative MRI and after AI-driven auto-contouring of MRI and ultrasound were 47 cc (IQR 34–65), 46 cc (IQR 35–63), and 44 cc (IQR 34–60), respectively. MRI targets were more frequently single (80%) and located in the posterior (61%) and equatorial (82%) regions of the prostate.

Table 2 shows the procedural and pathologic outcomes. Antibiotic prophylaxis consisting of cefixime, 400 mg once a day for 3 days, was administered in 146 patients (98%). The median device-time required from the import of MRI images to the fusion imaging was 5 min (IQR 3–6) and the median time to perform the biopsy was 15 min (IQR 12–17). Median VAS score at the end of the biopsy was 2 (IQR 2–4). Cancer detection rate was 64% overall and 56% for csPCa. PCa was detected in 35%, 65% and 84% of lesions scored as PI-RADS 3, 4 and 5, respectively. Considering MRI-targeted cores only, PCa and csPCa were found in 53% and 49% of cases, respectively. Outfield positive systematic cores were found in the same lobe of the target in 69% of cases, and in the contralateral lobe in 37% of cases. No significant difference was recorded in terms of detection rate between experienced and supervised novice users (61% vs. 66%, *p* 0.55).

## 4. Discussion

In recent years, evidence has accumulated in support of the execution of transperineal prostate fusion biopsies [1]. Despite the fact that there is still no consensus in favour of the software-guided fusion over the cognitive approach [4], two randomized controlled trials have shown increased detection rates of overall and clinically significant PCa when adopting a software-guided fusion in biopsy-naïve patients [2,3]. Other non-randomized studies showed a clear disadvantage in the cognitive approach when the MRI target diameter measures less than 1 cm [8,9].

To be effective and widely adopted, fusion biopsy devices should provide an accurate fusion imaging, but also be time-effective and user friendly. In this paper, we present our first series of transperineal fusion biopsies performed under local anaesthesia with the novel UroFusion system equipped with AI-driven auto-contouring. Several findings of this study deserve comment.

First, we showed that these procedures are quick, appearing in line with previous data reporting mean procedural duration for fusion biopsy of 15.9 ± 4.9 min [10]. The complete automatization of the fusion imaging part, with the auto-contouring of the prostate both on MRI and ultrasound, may speed up the procedure, making its duration comparable to that of a standard biopsy. We estimated a median of 5 min from the import of MRI DICOM images to the completion of fusion imaging; this time can even be reduced if the MRI images are already uploaded in the PACS system, which is unfeasible in our centre due to bureaucratic issues. While the median device-time of 5 min for fusion imaging is rapid, it is important to note that this study, being descriptive, does not allow for a direct comparison with other manual or semi-automated contouring devices in terms of time efficiency.

Second, the AI-driven auto-contouring provided excellent correlation between ultrasound and MRI volumes, with only 1 cc of median difference. This step, which to date was manually performed by the operator (as still happens for several other fusion biopsy devices), allows expert users to save time, and helps those less experienced in MRI reading to standardize the procedure and possibly improve their outcomes. In any case, the operator can intervene anytime to modify the contouring, if needed. With the recent advancements in the field of AI, several studies reporting promising results have evaluated the integration of AI in PCa management, including tasks relevant to biopsy such as prostate contouring and segmentation [5], diagnosis of csPCa or detection of the targets [11,12], and prediction of lymph node involvement [13]. To our knowledge, however, UroFusion is the first system to have been used in the routine clinical practice.

Third, we confirmed the good tolerability of TP fusion biopsy performed under local anaesthesia. The mean VAS score in this study was 3 at the end of the procedure, similar to a previous report showing mean numeric rating scale pain scores of 3.9 ± 2.1 at local anaesthesia administration and 3.1 ± 2.3 when performing biopsies [14]. We recommend to always wait a few minutes between the anaesthetic injection and the biopsy performance, to let the anaesthesia having its full effect. The AI-driven auto-contouring speeds up the procedure, thereby contributing to its tolerability; this may be particularly true for novice users.

Fourth, in the present study, the accuracy of the fusion biopsies performed with UroFusion was good in terms of cancer detection rate, comparable to other reports published in the literature. We found overall and csPCa detection rates of 35% and 23%, 65% and 61%, and 84% and 78% in lesions scored as PI-RADS 3, 4, and 5, respectively. In a recent meta-analysis, ISUP grade ≥ 2 PCa detection rates on a patient level were 20%, 55%, and 83% for PI-RADS 3, 4, and 5, respectively [15]. A previous multicentric study conducted by our group using a different fusion biopsy device reported overall and csPCa detection rates of 31% and 17%, 66% and 47%, and 89% and 79% in lesions scored as PI-RADS 3, 4, and 5, respectively [16]. While direct comparisons should be interpreted with caution due to potential differences in study populations and methodologies, our observed detection rates are broadly in line with those reported in the literature. Interestingly, in the present study, the detection rates were comparable between experienced users, with more than 500 fusion biopsy procedures done, and novice users, who performed the biopsy by themselves, under the supervision of an expert for obvious, ethical reasons. This reflects the fact that the UroFusion system appears user-friendly and easy to learn.

In our routine clinical practice, we perform our previously published technique of free-hand transperineal biopsy [7], which avoids the need for steppers or grids that are sometimes expensive and need to be disinfected and/or sterilized after each procedure. Furthermore, by doing free-hand biopsies we can choose the angle of insertion of our biopsy needle, avoiding pubic arch interference when sampling the anterior zone and reducing the number of skin punctures, with advantages in terms of pain and tolerability. The biopsy is done in-office, waiting for the patient to urinate before being discharged, with a fast patient turnover.

A final point to be discussed is related to the performance of systematic biopsies, which remain a key step of the procedure for the assessment of cancer volume and location. While the role of systematic biopsies in all biopsy-naïve patients is increasingly debated, in a per-lesion analysis, we confirmed our previous findings [17,18] that a non-negligible percentage of PCa is found outside MRI targets, in the same lobe in more than 60% of cases and in the contralateral lobe in more than 30% of cases. Most of these cancers are ISUP ≥ 2, so-called clinically significant. The detection of these cancer foci is essential for a correct treatment indication, particularly when offering a focal treatment or a hyperconservative surgery. Unfortunately, it would seem that MRI sees only the tip of the iceberg.

We acknowledge the limitations of this study, characterized by a small sample size without a comparative arm (e.g., manual contouring, other fusion systems, or cognitive biopsy) or a predefined sample size calculation. However, this is the first report about fusion biopsies performed with UroFusion AI-driven auto-contouring.

## 5. Conclusions

In this initial prospective experience, prostate fusion biopsies performed with UroFusion AI-driven auto-contouring system appeared time-efficient, user-friendly, accurate, and well tolerated, with comparable outcomes between experienced and novice users. Systematic biopsies remain highly recommended given the non-negligible rates of positive cores outside MRI targets.

## Figures and Tables

**Table 1:** Baseline characteristics.

Variable	*n* 148
Age, years, median (IQR)	69 (64–76)
PSA, ng/mL, median (IQR)	6.5 (3.9–9.1)
PSA density, median (IQR)	0.13 (0.06–0.18)
Positive familiar history, *n* (%)	19 (13%)
Biopsy naïve, *n* (%)	124 (84%)
Suspicious DRE, *n* (%)	50 (34%)
Suspicious extracapsular extension at MRI, *n* (%)	17 (11%)
Prostate volume at MRI, cc, median (IQR)	47 (34–65)
Prostate volume at MRI auto-contouring from MRI, cc, median (IQR)	46 (35–63)
Prostate volume at US auto-contouring, cc, median (IQR)	44 (34–60)
Difference in volume estimation between US and MRI auto-contouring, cc, median (IQR)	−1 (−4–2)
Number of targets, *n* (%) - single - multiple	118 (80%) 30 (20%)
Target diameter, mm, median (IQR)	11 (8–15)
PI-RADS score, *n* (%) - 3 - 4 - 5	26 (18%) 85 (57%) 37 (25%)
Target location, *n* (%) - Apex - Equator - Base - Posterior - Anterior - Transitional	43 (29%) 82 (55%) 30 (20%) 90 (61%) 18 (12%) 39 (26%)

Legend: DRE: digital rectal examination; IQR: interquartile range; PI-RADS: Prostate Imaging Reporting and Data System; US: ultrasound.

**Table 2:** Procedural and pathologic outcomes.

Variable	*n* 148
PROCEDURAL OUTCOMES	
Fusion biopsy operator, *n* (%) - Experienced - Supervised novice	54 (36%) 94 (64%)
Time needed for fusion imaging, min, median (IQR)	5 (3–6)
Time needed for biopsy, min, median (IQR)	15 (12–17)
Total numbers of cores taken, median (IQR)	15 (15–15)
Total number of positive cores, median (IQR)	3 (1–6)
VAS score at the end of the biopsy, median (IQR)	2 (1–3)
PATHOLOGIC OUTCOMES	
Cancer detection rate, *n* (%)	95 (64%)
ISUP grade, *n* (%) - 1 - 2 - 3 - 4 - 5	8/95 (8%) 50/95 (53%) 25/95 (26%) 9/95 (10%) 3/95 (3%)
Cancer detection rate at targeted cores only, *n* (%)	79 (53%)
ISUP grade at targeted cores only, *n* (%) - 1 - 2 - 3 - 4 - 5	6/79 (7%) 40/79 (51%) 25/79 (32%) 5/79 (6%) 3/79 (4%)
Cancer detection rate according to PI-RADS score, *n* (%) - 3 - 4 - 5	9/26 (35%) 55/85 (65%) 31/37 (84%)
ISUP ≥ 2 cancer detection rate according to PI-RADS score, *n* (%) - 3 - 4 - 5	6/26 (23%) 52/85 (61%) 29/37 (78%)
Cancer detection rate according to surgeon’s experience, *n* (%) - Experienced - Supervised novice	33/54 (61%) 62/94 (66%)
Outfield positive systematic cores, same lobe of the target, *n* (%) - ISUP ≥ 2	66/95 (69%) 59/95 (62%)
Outfield positive systematic cores in the contralateral lobe, *n* (%) - ISUP ≥ 2	35/95 (37%) 31/95 (33%)

Legend: IQR: interquartile range; ISUP: International Society of UroPathology; PI-RADS: Prostate Imaging Reporting and Data System; US: ultrasound; VAS: visual analogue scale.

## Data Availability

The data presented in this study are available on request from the corresponding author.
